# Treatment for Flexible Flatfoot in Children With Subtalar Arthroereisis and Soft Tissue Procedures

**DOI:** 10.3389/fped.2021.656178

**Published:** 2021-05-21

**Authors:** Bing Li, Wenbao He, Guangrong Yu, Haichao Zhou, Jiang Xia, Youguang Zhao, Hui Zhu, Tao Yu, Yunfeng Yang

**Affiliations:** Department of Orthopedics, Shanghai Tongji Hospital, Tongji University School of Medicine, Shanghai, China

**Keywords:** subtalar, surgical technique, flatfoot, treatment, pediatric

## Abstract

**Background:** Children with flexible flatfoot is common in clinics and there is no unified conclusion on surgical treatment. And for some patients with severe deformities, the correction of the subtalar joint arthroereisis combine the release of the Achilles tendon or gastrocnemius muscle release is still not satisfactory. The main aim of the present study was to investigate the therapeutic outcomes of subtalar arthroereisis combined with Achilles tendon or gastrocnemius recession and medial soft tissue (spring ligament, talonavicular joint capsule, tibionavicular ligaments and tibiospring ligaments) tightening for treating flexible flatfoot with severe deformities.

**Methods:** Thirty patients (32 feet) with pediatric flexible flatfoot who underwent subtalar arthroereisis and soft tissue procedures during January 2016 to January 2018. There were 18 males (20 feet) and 12 females (12 feet) with an average age of 9.5 years (range, 8–12 years). We used the AOFAS scores and VAS scores combined with angles measure to evaluate the pre-operative and post-operative status.

**Results:** Thirty patients (32 feet) were followed up for 25.3 months on average (range, 18–36 months). There was no infection. Post-operative foot pain, arch collapse, and other symptoms improved. At last follow-up, the Meary angle was decreased from 17.5° ± 4.4° to 4.1° ± 1.2° (*P* < 0.05), the talar-first metatarsal (AP) was decreased from 15.3° ± 3.1° to 4.8° ± 1.3°(*P* < 0.05), The mean AOFAS score was rose from 66.6 ± 5.8 to 88.6 ± 7.9 (*P* < 0.05), the mean VAS score was decreased from 6.6 ± 0.6 to 1.7 ± 0.3 (*P* < 0.05).

**Conclusion:** The subtalar arthroereisis combined with soft tissue procedures can effectively correct flexible flatfoot in children and it is a significant method for severe forefoot abduction reconstruction.

**Level of Evidence:** IV

## Introduction

Children flatfoot is common in clinics, most of which are flexible. Its etiology and pathological process are unclear (1). Most pediatric orthopedic surgeons believe that radiographic measurements such as Meary's angle, talonavicular coverage, the lateral talocalcaneal angle and the kite's angle, and the clinical assessments of foot, especially the forefoot, including foot flexibility, the quality and amount of forefoot pronation, triceps surae contracture, longitudinal arch and the valgus of the hindfoot, are very important for diagnosis (2–5). There are various treatments, including conservative and surgical methods. Conservative therapies include arch pads, orthopedic shoes, and braces (6–8). But like insole, the effectiveness of these conservative therapies remain uncertainty for pediatric flexible pes planus (9, 10). Surgical procedures include soft tissue surgeries and bone surgeries including lateral column lengthening, medializing calcaneal osteotomy and arthroereisis (11, 12). However, there is no unified conclusion on surgical treatment. Although osteotomies can correct the forefoot abduction, they are challenging to operate and increase the probability of complications such as fracture malunion, delayed union or non-union, epiphyseal injuries, etc. (13–18).

Calcaneo-stop procedure was reported by Alvarez in 1995, which used a extra-articular screw to achieve arthroereisis, but it could make joint stiffness. In recent years, with the improving understanding of flexible flat feet and the development of the subtalar joint arthroereisis, which can allow a certain range of motion. It is gradually accepted by orthopedic surgeons. Although sinus tarsi pain were reported as the most frequent complication of subtalar arthroereisis, this procedure can effectively correct the calcaneal valgus and raise the talar head without damaging the bones and has been reported as a minimally-invasive, effective and low-risk procedure in the treatment of flatfoot mainly in children but also in adults (1, 19–23). Simultaneously, the tension of the calf triceps is an essential cause of the arch collapse. Therefore, the release of the gastrocnemius muscle or Achilles tendon can effectively improve the cavus deformity and reduce the stress in the mid-forefoot.

However, for some patients with severe deformities, the correction of the subtalar joint arthroereisis combine the release of the Achilles tendon or gastrocnemius muscle release is still not satisfactory. The main aim of this study was to investigate the therapeutic outcomes of subtalar arthroereisis combined with Achilles tendon or gastrocnemius recession and medial soft tissue (spring ligament, talonavicular joint capsule, tibionavicular ligaments and tibiospring ligaments) tightening for treating flexible flatfoot with severe deformities.

## Materials and Methods

### Patients and Material

Thirty children with flexible flat feet (32 feet) were included in this study. There were 18 males (20 feet) and 12 females (12 feet) with an average age of 9.5 years (range, 8–12 years). All the patients had a medial longitudinal arch collapse, pain, hindfoot eversion, walking difficulties and prominent forefoot abduction. The weight-bearing AP and lateral X-ray, arch height, Meary angle, and talo-first metatarsal angle were measured pre-operatively. The foot function was evaluated pre-operatively with American Ankle and Surgery Association (AOFAS) ankle and hindfoot scoring and VAS score. The Silverskold test was used to evaluate the gastrocnemius or Achilles tendon contracture. The testing method was to maintain the posterior foot in a neutral position and the knee to be fully straightened. Improved ankle dorsiflexion with knee flexed suggesting the presence of the gastrocnemius contracture; equivalent ankle dorsiflexion with knee flexion and extension suggesting the presence of the Achilles contracture. All 32 feet had severe forefoot abduction with gastrocnemius or Achilles tendon contracture.

All patients which were included in the present study were gave written informed consent for their data in this study. All data was obtained from the clinical and radiograph records. This study was approved by the Institutional Review Board/Ethics Committee of the authors' institution.

The Talar-Fit implant of Meditec used in this study has anatomy that completely mimics the tarsal sinus, which can avoid discomfort and pain caused by activity. Its blunt threads can reduce the incidence of soft tissue irritation and synovitis. Furthermore, deep threads can promote soft-tissue growth and achieve further stability. Last but not least, the procedure is simple, and it is not necessary to cut the interosseous ligament, which is more conducive to the stability of the subtalar joint.

From January 2016 to January 2018, 30 children with flexible flat feet were treated with subtalar arthroereisis combined with gastrocnemius muscle or Achilles' tendon release. Moreover, all feet in this study were performed the tightening procedure, which tightens the spring ligament, talonavicular joint capsule, and the superficial layer of the deltoid ligament (tibionavicular and tibiospring ligaments). The efficacy was satisfactory.

### Inclusion and Exclusion Criteria

#### Inclusion Criteria

Aged 8–12 years, flexible flat feet, arch collapse, hindfoot valgus, gastrocnemius or Achilles tendon contracture, severe forefoot abduction.

#### Exclusion Criteria

Rigid flat feet, flat feet combined with other foot deformities (such as the tarsal coalition, rheumatoid joint disease, muscle imbalance caused by cerebral palsy or other neuropathies, overcorrection of talipes cavus).

This study was approved by the Institutional Ethics Committee of the authors' institution (approval number: K-W-2020-003).

### Surgical Procedures

All operations were performed by one chief surgeon of foot and ankle surgery, and his assistants were attending surgeons. And the chief surgeon has more than 50 cases of flatfoot orthopedic surgery experience for adults or children. The surgery was performed under the epidural anesthesia. The thigh tourniquet was used. Firstly, the Silverskold test was performed. For gastrocnemius release, the posterior-medial incision at the lower middle calf was used, ~2–3 cm. The gastrocnemius tendon-abdominal joint was exposed, followed by cutting off the tendon part sharply to make the ankle dorsiflexion >10° when the knee was in extension. Attention should be taken to protect the sural nerve. For Achilles tendon lengthening, a 4 cm medial incision was made and the “*Z*-shape” lengthening was performed to make the ankle dorsiflexion > 15° when the knee was in fully extension. Then the subtalar arthroereisis was performed. The hip on the ipsilateral side was uplifted by sandbag to avoid excessive external rotation of the foot, conducive to the exposure of the sinus tarsi. A longitudinal 2 cm incision was made under the lateral malleolus tip, at the body surface projection of sinus tarsi. Pay attention to protect the branches of the superficial nerve and avoid damage to the talus and calcaneus's interosseous ligament. Followed cutting the fascia layer, the phalangeal extensor muscles were pulled distally. The stripper was inserted into the tarsal sinus along the talar neck after the full exposure of the tarsal sinus. The talus head was raised upward and rearward to restore its physiological position and correct the pronation of the hindfoot (24). A guidewire was inserted into the tarsal sinus in the direction of the tunnel. After confirming the guidewire position with C-arm, the trial model was plugged along the guidewire from small to large. The weight-bearing AP, lateral, and calcaneal axial perspectives were taken after each implantation to evaluate the positional relationship of the mid-hind foot bones, joint motion and the implant position until the appropriate size was determined. If the size is too small, the deformity correction is not sufficient, and the implantation loosening can occur. If the size is too large, the deformity is overcorrected, and it may result in persistent pain of the tarsal sinus. The lateral edge of the implant should overlap with the talus's lateral edge, but the medial edge of the implant should not exceed the midline of the talus neck (25). Satisfactory corrections: the talus axis is almost consistent with the first metatarsal axis in lateral film (<5°).

And if there is still remaining forefoot abduction, a medial talonavicular incision should be performed to expose and cut tibionavicular ligaments, tibiospring ligaments, talonavicular joint capsule and spring ligament layer-by-layer. Then tighten the ligaments and joint capsule by plicating suture. Satisfactory corrections: the talus axis is almost consistent with the first metatarsal axis in AP (<5°).

### Post-operative Care

**Figure 1:**
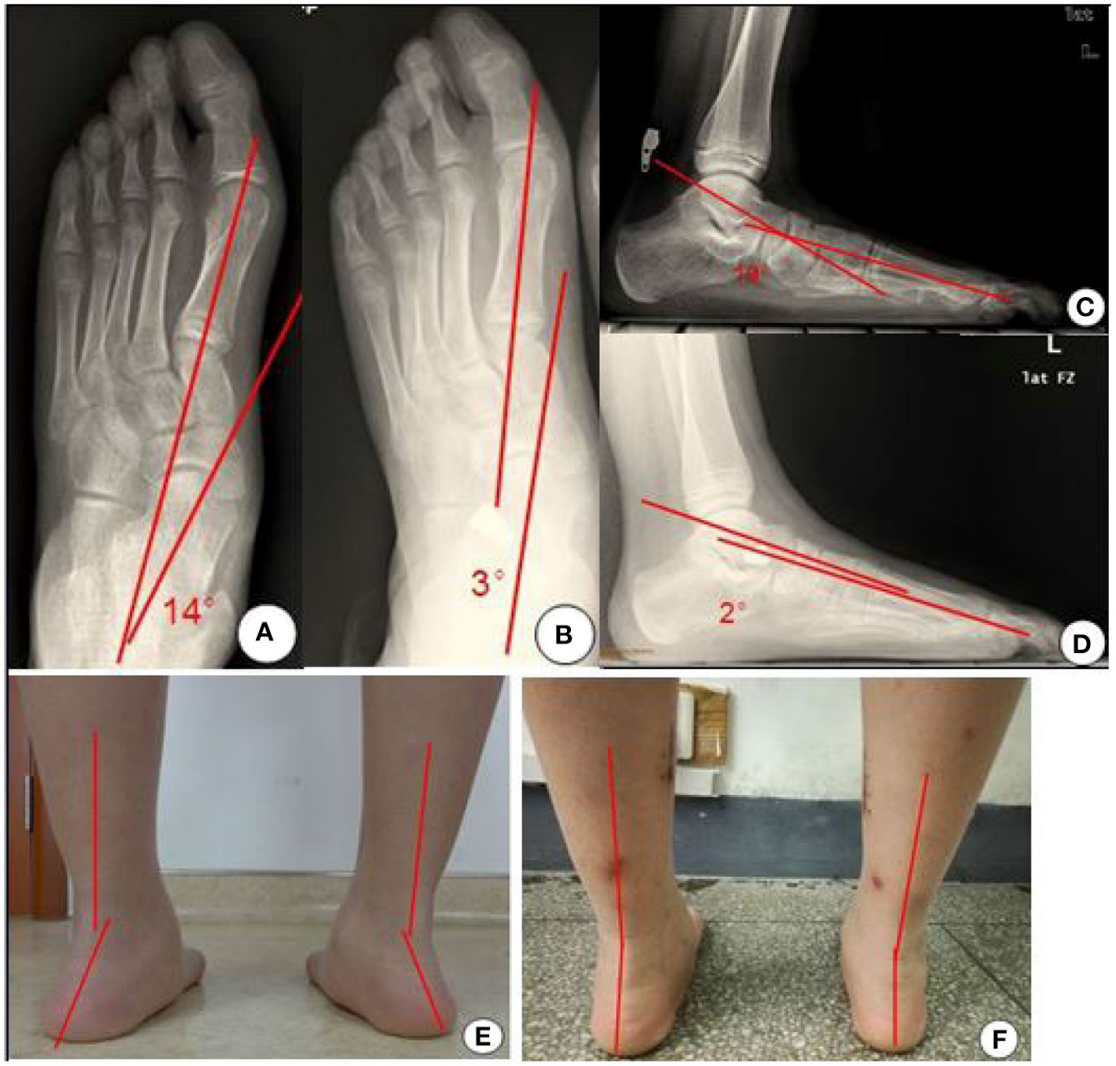
X-ray films and appearance photos before and after operation. **(A)** The left foot X-ray film (AP) before operation. **(B)** The left foot X-ray film (AP position) after operation. **(C)** The lateral X-ray film of the left foot before operation. **(D)** The lateral X-ray film of the left foot after operation. **(E)** The posterior foot line of force before operation. **(F)** The posterior foot line of force after operation.

Patients underwent Achilles tendon lengthening and medial soft tissue tightening require plaster fixation for 4 weeks. Plaster fixation is not required for patients without soft tissue surgery or gastrocnemius release. They wear walking braces at night and perform passive plantar and dorsal flexion exercises on the first day after surgery. The active flexion exercise of the ankle was performed on the second day after surgery. The suture was removed 12–14 days after the operation. Non-weight-bearing exercises, including swimming and bicycling, were usually recommended 3 weeks after surgery. The partial weight-bearing functional exercise was started 6 weeks after surgery. Immediately, 6-week and 12-week post-operative AP, lateral, and oblique radiographs were performed. The X-ray was taken every 3 months after 12 weeks to observe the correction of the deformity and the position of the implant (Figure 1). At the last follow-up, the Meary angle and the talus-first metatarsal angle were recorded to compare with the pre-operative data. The function was evaluated by AOFAS ankle-hind foot score and VAS score (26). All of these patients returned to sports within 12 months.

### Statistical Analysis

SPSS 17.0 statistical software package was used for analysis. The results were presented as the mean and standard deviation. The angles, VAS scores, and AOFAS scores before and after surgery were compared between groups. The paired *t*-test was used. Significance was set at *P* < 0.05.

## Results

Thirty patients(32 feet) were followed up for 18–36 months, with an average of 25.3 months. All of the 30 children underwent conservative treatment using the insole before undergoing surgery. All feet in this study were performed the tightening procedure, 24 feet underwent gastrocnemius release and four feet underwent gastrocnemius release. All operations were performed by one chief surgeon of foot and ankle surgery, and his assistants were attending surgeons. All incisions healed in the first stage without infection. The implant position was lost in one case due to excessive activities and sprain 3 months after surgery. Reoperation implantation of the subtalar joint arthroereisis was performed. During the follow-up, no complications such as loosening of the implant and recurrence of flat foot deformity occurred in other cases. The pain was significantly relieved, and arch collapse was significantly improved (Figure 1). At the last follow-up, the Meary angle improved from an average of 17.5° ± 4.4° pre-operatively to 4.1° ± 1.2° post-operatively (*P* < 0.05). The talus- first metatarsal angle of AP improved from an average of 15.3° ± 3.1° before surgery to 4.8° ± 1.3° after surgery (*P* < 0.05). The AOFAS ankle-hindfoot score increased from an average of 66.6 ± 5.8 pre-operatively to 88.6 ± 7.9 post-operatively (*P* < 0.05 The VAS score improved from an average of 6.6 ± 0.6 before surgery to 1.7 ± 0.3 after surgery (*P* < 0.05) (Table 1). All of these patients returned to sports within 12 months.

**Table 1:** Radiographic comparison between pre-operative and last follow-up values.

	**Pre-operative**	**Post-operative**	***P-*value**
Talo-first metatarsal angle in AP (°)	15.3 ± 3.1	4.8 ± 1.3	<0.0001
Talo-first metatarsal angle in lateral view (°)	17.5 ± 4.4	4.1 ± 1.2	<0.0001
AOFAS score	66.6 ± 5.8	88.6 ± 7.9	0.0002
VAS-FA score	6.6 ± 0.6	1.7 ± 0.3	0.0002

## Discussion

In 1946, Chambers first proposed using autologous bone implantation into the tarsal sinus to limit the excessive eversion of the subtalar joint. In 1952, Grice applied subtalar joint fusion and bone grafting into tarsal sinus for children with flatfoot. Post-operative complications such as degenerative arthritis of the adjacent joints and the walking difficulty on uneven roads occurred. Subsequently, Haraldsson and Lelivre modified the above methods and pointed out that it is essential to limit the subtalar joint's excessive valgus without the joint fusion. Since then, the concept prototype of the subtalar joint arthroereisis came into being. The ROM (range of motion) of the subtalar joint is greater in flexible flatfeet compared with healthy feet. Excessive valgus of the subtalar joint can cause the collapse of the medial foot arch, lateral subluxation of the talonavicular joint, and abduction of the forefoot, resulting in the inflammatory pain of the posterior tibial tendon insertion point, and the laxity of talonavicular joint capsule and spring ligament. Therefore, the symptoms of flexible flat feet are mainly medial pain, activity difficulty, inability to walk on uneven roads, and painful cramps. Most children can experience recovery with age, and only a small proportion of people have persistent symptoms. Surgery is indicated when they meet four or more of the following criteria (24). ① No clinical or radiographic improvement after 2-year conservative treatment; ② Valgus angle of hindfoot > 10°; ③ Achilles tendon contracture; ④ Viladot footprint type II, III, or IV; ⑤ Meary angle < 10°; ⑥ Moreau-Costa- Bartani Angle > 130° or Kite angle > 25°. Besides, age is also an essential factor for surgery (27). Most children can recover themselves before 8 years old, and surgery is not suitable for them. One purpose of subtalar joint arthroereisis is to reshape the bones and ligaments of the hindfoot during children's development. This process takes at least 2 years, and the remodeling time is not sufficient if the patient is more than 12 years. Therefore, surgery is indicated for children of 8 to 12 years. The patients in this study were 8–12 years old, and all met the above-mentioned surgical indications.

The choice of the implant size is very crucial. The principle is the minimum size that can correct the deformity and can be stably maintained in the tarsal sinus when activity. The implant should maintain the calcaneus at 2~4° eversion position, which can be determined by the AP, lateral, oblique, and calcaneal axial X-ray. The tail of the implant should be consistent with the lateral edge of the talus on the AP X-ray, and its head should not exceed the midline of the talus neck. There are no specific normative standards for the Talar-Fit implant of Meditec and we think this study can be used as a reference. It remains controversial whether the fatty tissue in the tarsal sinus needs to be removed before implantation. Some surgeons believe that it is necessary to remove the fatty tissues richly supplied with nerve endings to avoid the pain caused by implant simulation (28). Some experts have pointed out that the cause of the pain may be related to the improper position or size of the implant (29). Therefore, some surgeons do not remove soft tissues. They believe that the thread-like structure of the implant can be inosculated with the soft tissue to maintain stability. The firmness may be affected if the soft tissue is removed, and the implant directly contacts the hard bone (30).

In patients with triceps contracture, the traction force increases, and heel stress decreases when walking. Furthermore, the stress transmitted forward through the ankle joint increases, leading to the medial longitudinal arch and metatarsal head stress increase. Therefore, the triceps does not affect on the maintenance of the arch, but its contracture is an essential cause for the arch collapse. Some scholars have found that the tension of the Achilles tendon increases after the implantation through biomechanical studies, and pointed out that the tendency of a cavus foot will be more visible even there is no cavus foot before surgery (31). Therefore, the gastrocnemius muscle or Achilles tendon release should be performed if there is a contracture. This procedure can correct the cavus deformity and improve the calcaneus eversion and forefoot supination.

Severe children flexible flatfeet have not only arch collapse and hindfoot valgus, but also talonavicular subluxation and abduction of the forefoot. For this deformity, subtalar joint arthroereisis alone cannot correct the deformity well. The traditional methods are mainly lateral extension osteotomy, such as Evans osteotomy or arthrodesis. Chou and Halligan (32) reported that treatment of severe, painful pes planovalgus with hindfoot arthrodesis and wedge-Shaped tricortical allograft were reliable and could provide satisfactory correction of deformity, but arthrodesis could accelerate the degeneration of adjacent joints, so it should be used as the final treatment. Kumar and Sonanis (17) made a systematic review of the literature on lateral column lengthening in the treatment of children's pes planovalgus. The results showed that the incidence of complications of osteotomy was as high as 17.5% (18/103), including nerve related, pseudo joint, bone non-union and metal related complications accounted for 17.5% (18/103). For some specific cohorts' outcomes, there were some articles addressing the body weight effects on implant extrusion after the procedure that a higher BMI is related to implant extrusion and worse results after subtalar arthroereisis. Surgery improved the participation in physical activities as well as the emotional status and footwear issues (33–36). These studies have paid little attention to the importance of medial foot ligament structures, so if there is still significant forefoot abduction after subtalar arthroereisis, the medial spring ligament, the talonavicular capsule, and the superficial deltoid ligament tightening are indicated. The combined procedure can effectively correct remaining forefoot abduction and restore the mid-forefoot alignment. The 30 patients in this study did not have the above serious complications. Compared with the traditional osteotomy, the operation is straightforward, and the probability of post-operative complications is also reduced.

For flexible children flatfeet with accessory navicular bone, we did not perform any other extra procedure. After the deformity is corrected, the alignment is restored, and the stress at the posterior tibial tendon stop and accessory navicular bone will be significantly reduced, which is conducive to the disappearance of inflammatory reactions. Also, for children patients, the accessory navicular bone still can fuse with the navicular bone. Therefore, we did not address the accessory navicular bone in 12 cases, and there were no apparent symptoms after surgery.

The limitations of this study include the small sample size, and clinical application as well as longer term follow-up is required to demonstrate the clinical usefulness of this procedure.

## Conclusion

In short, the treatment principle of flexible children flatfeet is to correct deformities and restore the alignment. The subtalar joint arthroereisis has the advantages of simple, minimally invasive, and rapid recovery. It effectively corrects deformities, restores the alignment, and reconstruct the functions of the soft tissue without joint fusion. This technology has a promising application prospect with the development and improvement of the subtalar joint implant. However, subtalar joint arthroereisis cannot be applied blindly, and surgical indications and contraindications should be kept in mind. More importantly, for some severe forefoot abduction cases, when the correction of the subtalar joint arthroereisis is not satisfactory, it is necessary to combine the release of the Achilles tendon or gastrocnemius muscle release, the spring ligament and deltoid ligament this technology has a better application prospect. Individualized surgery can better correct the deformity, restore the alignment, achieving more commendable clinical results.

## Data Availability Statement

The original contributions presented in the study are included in the article/supplementary material, further inquiries can be directed to the corresponding authors.

## Ethics Statement

The studies involving human participants were reviewed and approved by Shanghai Tongji Hospital Ethics Committee. Written informed consent to participate in this study was provided by the participants' legal guardian/next of kin.

## Author Contributions

BL, WH, and YY: study design and development. GY, HZho, and JX: data extraction and first draft. WH, YZ, HZhu, TY, and YY: data analysis and interpretation. All authors contributed to critical revision of content and read and approved the final version.

## Conflict of Interest

The authors declare that the research was conducted in the absence of any commercial or financial relationships that could be construed as a potential conflict of interest.
